# Establishment of Nested PCR for the Detection of *Pseudomonas plecoglossicida* and Epidemiological Survey of *Larimichthys crocea* in the Southeast Coastal Region

**DOI:** 10.3390/ani14101427

**Published:** 2024-05-10

**Authors:** Xinbing Duan, Jiji Li, Hui Shi, Zhen Tao, Xuelian Wei, Yingying Ye, Baoying Guo

**Affiliations:** 1 Marine Science and Technology College, Zhejiang Ocean University, Zhoushan 316022, China; duanxinbing@zjou.edu.cn (X.D.); guobaoying@zjou.edu.cn (B.G.); 2 National Engineering Research Center for Marine Aquaculture, Zhejiang Ocean University, Zhoushan 316022, China; weixl1247@163.com (X.W.); yeyy@zjou.edu.cn (Y.Y.); 3 Key Laboratory of Mariculture and Enhancement of Zhejiang Province, Zhejiang Marine Fisheries Research Institute, Zhoushan 316021, China; 4 School of Fishery, Zhejiang Ocean University, Zhoushan 316022, China; taozhenlx@gmail.com

**Keywords:** *Pseudomonas plecoglossicida*, nested PCR, visceral white nodules disease, *Larimichthys crocea*

## Abstract

**Simple Summary:**

In this paper, a three-round nested PCR assay with high specificity and sensitivity (2.62 copies/reaction), employing the *Pseudomonas plecoglossicida sctU* gene, was established. The detection system was used to detect large yellow croaker samples in the Zhejiang and Fujian areas. It was found that March and April were the peak times of visceral white nodules disease, which was inversely related to temperature. Using this detection method, early detection of visceral white nodules disease in large yellow croakers could be realized.

**Abstract:**

The visceral white nodules disease in the internal organs of *Larimichthys crocea* has caused significant harm in the aquaculture of this species, with *Pseudomonas plecoglossicida* considered one of the core pathogens causing this disease. In this study, we designed three pairs of specific nested PCR primers targeting the *sctU* gene of *P. plecoglossicida*, a crucial component of the Type III secretion system (T3SS), which is instrumental in bacterial pathogenesis and virulence. Through the optimization of PCR reaction conditions, specificity testing, and sensitivity determination, a method was established for the accurate detection of *P. plecoglossicida*. This method yielded single amplification products, exhibited a false positive rate of zero for reference bacteria, and achieved a detection sensitivity of a minimum of 2.62 copies/reaction for the target sequence. Using the detection method, we conducted analyses on the diseased populations of *L. crocea*, involving a total of 64 screened fishes along the southeast coast of China from 2021 to 2023. The results revealed that the infection rate of *P. plecoglossicida* in diseased *L. crocea* exceeded over 90% in March and April, while in other months, the maximum recorded infection rate was merely 10%. The detection method developed in this study shows potential for early warning and routine monitoring of visceral white nodules disease in the internal organs of species such as *L. crocea*.

## 1. Introduction

The large yellow croaker (*Larimichthys crocea*), a renowned marine economic fish species in China, is highly esteemed for its organoleptic properties and abundant nutritional content [1]. Following significant advancements in *L. crocea* breeding techniques [2], rapid growth has been observed in the aquaculture industry of this species, particularly in Zhejiang, Fujian, and Guangdong, its primary cultivation regions. In recent years, as the farming scale has expanded, the aquaculture environment has gradually deteriorated, leading to frequent disease outbreaks among large yellow croakers reared in high-density cages. These diseases continue to have a profound impact on the growth, metabolism, digestion, and immune system of the fish [3,4]. One of the most devastating bacterial diseases affecting large yellow croakers is visceral white nodule disease (VWND). This chronic infectious disease is characterized by the appearance of numerous white nodules, ranging from 1 mm to 2 mm, in organs such as the spleen and kidney, accompanied by intestinal inflammation [5]. The high incidence and infectivity of this disease pose a significant threat to *L. crocea* populations [6]. *Pseudomonas plecoglossicida* has been identified as one of the primary pathogens responsible for causing VWND in large yellow croakers [6,7,8]. It is crucial to understand the pathogenesis and develop effective control strategies for this bacterium in order to safeguard the health and sustainability of *L. crocea* aquaculture.

Currently, the development of an effective treatment or prophylactic drug for VWND remains elusive, with significant delays [9]. The early detection of the disease is challenging, significantly compromising the effectiveness of treatments administered after the appearance of white nodules on the internal organs. Studies have proposed that antibiotics can be used to treat VWND [10,11], but it was found that the treatment effect is not good in practical application. At the same time, the abuse of antibiotics will not only cause drug resistance in bacteria but also cause great damage to the marine environment. Sun [12] has screened two sensitive drugs of *P. plecoglossicida* from large yellow croakers for early intervention in VWND. This strategy highlights the significance of quickly beginning pharmacological interventions as soon as *P. plecoglossicida* infection is detected in fish, which has been shown to provide substantial therapeutic advantages. This underscores the crucial significance of early monitoring in the management of VWND in large yellow croakers.

Studies have found that the predominant pathogen causing VWND in large yellow croakers is *P. plecoglossicida* [13,14]. As an opportunistic pathogen, the infection in fish is intricately linked to temperature [15,16]. *P. plecoglossicida* notably encodes both the type III (T3SS) and type VI secretion systems (T6SS) [17,18], which are key protein secretion mechanisms common among Gram-negative pathogenic bacteria. These systems serve as crucial avenues for bacterial virulence factors to exert their deleterious effects [19]. Several scholars have found that T3SS can provide a channel for Gram-negative bacteria to directly secrete and inject effector proteins into the cytoplasm of host cells to exert virulence functions [20,21]. The *sctU* gene encodes a critical component of the Type III secretion system (T3SS). The protein synthesized by this gene plays a pivotal role in forming the export apparatus of the T3SS. Additionally, Kuhlen et al. [22] have identified the export gate as a crucial element in the secretion channel. Nested PCR (Nested Polymerase Chain Reaction) is a gene amplification technology in vitro established by Mullis and Faloona [23] in 1987 on the basis of conventional PCR. This method utilizes two sets of primers: an outer primer and an inner primer. To enhance the detection of *P. plecoglossicida* in low-concentration samples, we employed a triple-nested PCR method known for its high sensitivity and specificity compared to conventional PCR methods. Nested PCR can be used to identify the positive samples, specifically when the content of the template DNA is very low [24].

In this study, based on the sequence of the *sctU* gene in the complete genome of *P. plecoglossicida* in NCBI, three pairs of specific nested PCR primers were designed, adhering to the principles of high intraspecies conservation and interspecies specificity. We aim to develop an efficient, sensitive, and accurate nested PCR detection technique that will provide essential technical support for monitoring.

## 2. Materials and Methods

### 2.1. Strains Tested

The *P. plecoglossicida* XSDHY-P (CP031146) was provided by the Disease Control Laboratory, College of Fisheries, Zhejiang Ocean University. *Pseudomonas putida* (BNCC192818), *Pseudomonas fluorescens* (BNCC336632), *Pseudomonas aeruginosa* (BNCC125486), *Aeromonas hydrophila* (BNCC337115), *Vibrio parahaemolyticus* (BNCC333072), *Vibrio harveyi* (BNCC336937), *Vibrio alginolyticus* (BNCC337013), *Vibrio fluvialis* (BNCC337051), and *Vibrio vulnificus* (BNCC186281) were purchased from the BeNa Culture Collection (Kunshan, China).

### 2.2. Experimental Methods

#### 2.2.1. Bacterial Activation Culture and DNA Extraction

After the lyophilized bacterial powder was completely dissolved, 200 μL bacterial suspension was aspirated and evenly spread onto the plate. All strains were incubated at 220 rpm/min for 16 h at constant temperature. Except for *P. plecoglossicida* and *V. parahaemolyticus*, which were cultured at 37 °С, the cultivation temperature of the other bacteria was 30 °С. Subsequently, single colonies that had been cultured overnight were selected and propagated in a liquid medium. After the completion of the expansion culture, bacterial DNA was extracted using two methods: the boiling water bath method [25] and the improved salting-out method [26]. The extracted DNA was then stored at −20 °С.

#### 2.2.2. Pathological Observation

Diseased large yellow croakers for pathological analysis were collected from marine culture cages in Ningde, Fujian Province. These fish measured 32 ± 4 cm in length, 9 ± 1 cm in width, and weighed 400 ± 80 g. While most exhibited no external wounds, some displayed surface ulcers. We used optical microscopy to detect internal and external parasites. The diseased tissue, dissected on site, was sectioned into 1 cm^3^ pieces, preserved in 4% paraformaldehyde, and processed into pathological sections after 24 h of soaking.

#### 2.2.3. Primer Design and Synthesis

For the detection of *P. plecoglossicida*, we utilized a triple-nested PCR approach. Initially, the outer primers are combined with the template DNA to perform the first round of amplification. Subsequent rounds utilize progressively internal primers, enhancing the amplification specificity and yielding a high concentration of target DNA from the minimal starting material. This method substantially increases detection accuracy, offering about a 100-fold improvement over traditional PCR techniques. Based on the *sctU* gene in the full-length sequence of *P. plecoglossicida* XSDHY-P (CP031146) in GenBank, specific sites were identified through MEGA alignment, and three pairs of nested primers were subsequently designed and synthesized by Qingke Biological Company (Shanghai, China). The details are shown in Table 1.

#### 2.2.4. Construction of Nested PCR System

The distinct annealing temperatures for the amplification primers were set at 50 °C, 52 °C, 54 °C, 56 °C, and 58 °C, with the extension time established at 90 s based on the predicted product length. The reaction program for the first round of amplification included pre-denaturation at 94 °C for 4 min, denaturation at 94 °C for 30 s, annealing at 52 °C for 30 s, extension at 72 °C for 1 min, followed by 30 cycles, and a final extension at 72 °C for 5 min. In the second round of amplification, products of the first round were used as templates, and the reaction program consisted of pre-denaturation at 94 °C for 4 min, denaturation at 94 °C for 30 s, annealing at 54 °C for 30 s, extension at 72 °C for 30 s, 30 cycles, and a final extension at 72 °C for 5 min. The third round of amplification used the products of the second round of amplification as templates, with a reaction program of pre-denaturation at 94 °C for 4 min, denaturation at 94 °C for 30 s, annealing at 52 °C for 30 s, extension at 72 °C for 30 s, 30 cycles, and a final extension at 72 °C for 5 min. PCR products were examined by electrophoresis on a 1% agarose gel after the third round of PCR amplification.

#### 2.2.5. Tests of Specificity

According to the nested PCR reaction steps constructed in this study, *P. plecoglossicida*, *P. putida*, *P. fluorescens*, *P. aeruginosa*, *A. hydrophila*, *V. parahaemolyticus*, *V. harveyi*, *V. alginolyticus*, *V. fluvialis*, and *V. vulnificus* were simultaneously amplified and detected, and the specificity of the nested PCR method was evaluated.

#### 2.2.6. Detection of Sensitivity

The original DNA concentration of *P. plecoglossicida* was determined using NanoDrop 2000, and the copy number of the plasmid was calculated according to the following formula: N = (C × NA)/M. The DNA of *P. plecoglossicida* was diluted by a 10^−1^–10^−10^ dilution gradient and then amplified according to the nested PCR system, and the sensitivity of the nested PCR method was evaluated.

#### 2.2.7. Construction of Positive Control Plasmids

The P.ple-sctU-O group primers were used to amplify the template, and the PCR products were analyzed by electrophoresis on a 1% agarose gel. Subsequently, the purified PCR fragments were ligated into the pMD19-T vector and transformed into DH5α competent cells. The transformed competent cells were then plated onto LB solid medium containing ampicillin, X-Gel, and IPTG and incubated for 12 h at 37 °C to facilitate blue–white colony screening. Single white colonies were picked and cultured in a 5 mL LB liquid medium for expansion, and plasmids were extracted using a plasmid extraction kit after overnight culture.

#### 2.2.8. Sample Collection and DNA Extraction of Large Yellow Croaker

From September 2021 to May 2023, a total of 64 large yellow croakers were collected from Zhoushan, Ningde, and Ningbo City. The muscle, surface mucus, gills, spleen, and kidneys were dissected on the spot and stored in sterile tubes at −80 °С. The tissue samples and surface mucus DNA of a large yellow croaker were extracted by the salting out method for subsequent sample detection experiments. A total of 192 samples were collected, of which 164 samples were successfully extracted with DNA, with a success rate of 85.4%, and the detailed information is shown in Table 2.

## 3. Results

### 3.1. Observation of Visceral White Nodules Disease in Large Yellow Croaker

**Figure 1:**
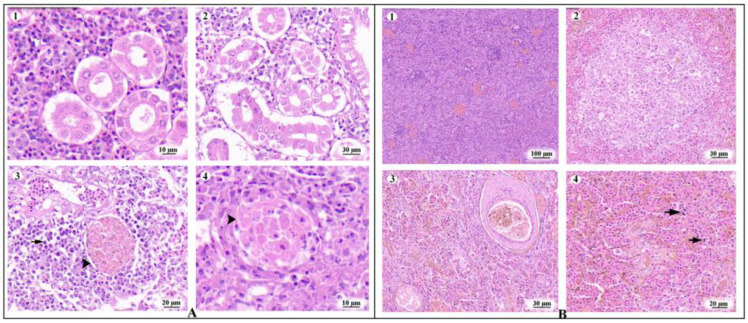
Pathological changes in the spleen and kidney tissues of diseased fish. (**A**) Pathological changes in the kidney tissues of diseased *L. crocea*. (1) Healthy kidney of yellow croaker; (2) Glomerular disintegration, tubular lumen obstruction; (3) White blood cell infiltration at the arrowhead; (4) The visible “white nodules” at the arrowhead. (**B**) Pathological changes in the spleen tissues of diseased *L. crocea*. (1) Healthy spleen of yellow croaker; (2) Blurred boundaries of splenic parenchymal cells, vacuolization; (3) Spleen nodule; (4) Neutrophil infiltration at the arrowhead.

**Figure 2:**
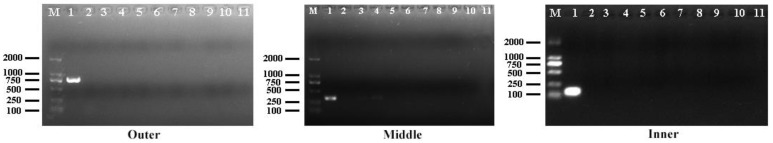
Specificity testing. M: DL 2000 DNA Marker, 1–11: *P. plecoglossicida* DNA, *P. putida* DNA, *P. aeruginosa* DNA, *P. fluorescens* DNA, *A. hydrophila* DNA, *V. harveyi* DNA, *V. parahaemolyticus* DNA, *V. alginolyticus* DNA, *V. fluvialis* DNA, *V. vulnificus* DNA, and ddH_2_O.

**Figure 3:**
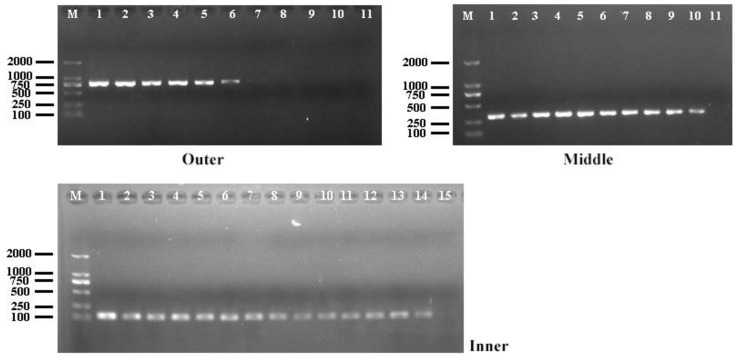
Sensitivity testing. M: DL 2000 DNA Marker, 1~15: DNA content per reaction, 1: 2.62 × 10^10^ copies, 2: 2.62 × 10^9^ copies, 3: 2.62 × 10^8^ copies, 4: 2.62 × 10^7^ copies, 5: 2.62 × 10^6^ copies, 6: 2.62 × 10^5^ copies, 7: 2.62 × 10^4^ copies, 8: 2.62 × 10^3^ copies, 9: 2.62 × 10^2^ copies, 10: 26.2 copies, 11: 13.1 copies, 12: 6.55 copies, 13: 3.28 copies, 14: 2.62 copies, 15: 1.31 copies.

**Figure 4:**
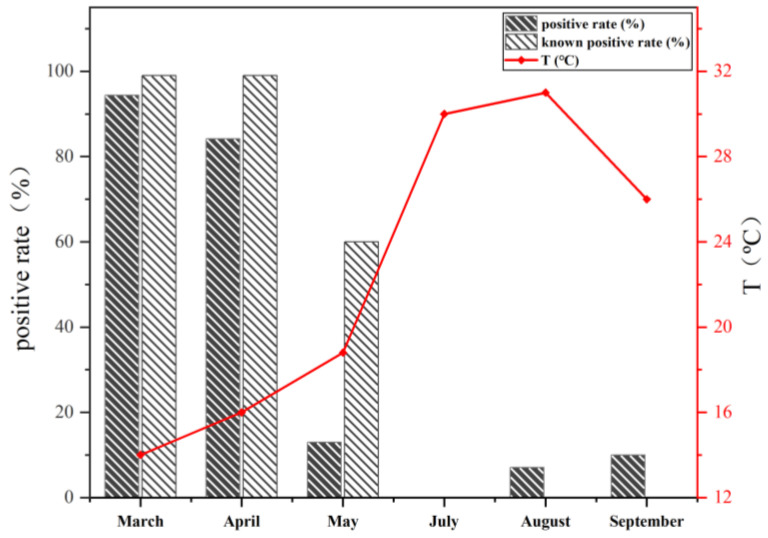
Positive rates of *P. plecoglossicida* in large yellow croaker samples in different months.

There were no wounds on the body surface of the diseased large yellow croakers, and a large number of white nodules were found in the spleen and kidney of the diseased fish. Additionally, some diseased large yellow croakers exhibited enlarged livers and abdominal fluid accumulation. To further investigate these pathological changes, the spleen and kidneys of these diseased fish were paraffin-embedded and analyzed using HE staining. Figure 1A shows the HE-staining pathological section of the kidney. Figure 1A—(1) is the healthy large yellow croaker kidney; in Figure 1A—(2), the glomeruli were disintegrated, and the lumen of the renal tubules was occluded and deformed. The pathological section highlighted by the arrow demonstrates significant white blood cell infiltration (Figure 1A—(3)), which indicates the presence of inflammation. In Figure 1A—(4), the bacteria were surrounded by tissue and appeared as pathological nodules, which were the macroscopic white dots. Figure 1B shows the HE staining pathological section of the spleen, and Figure 1B—(1) shows the healthy large yellow croaker spleen tissue. In Figure 1B—(2), the splenic parenchymal cells exhibit blurred boundaries and numerous vacuoles. Figure 1B—(3) reveals the presence of pathological nodules, corresponding to the visible “white nodules” along with tissue amyloidosis. The presence of neutrophil infiltration in Figure 1B—(4) indicates the presence of inflammation.

### 3.2. Tests of Specificity

According to the established reaction conditions, all strains and the negative control group were subjected to amplification and detection. The results showed that only *P. plecoglossicida* yielded bands in three rounds of amplification, while other groups tested negative, demonstrating good specificity (Figure 2).

### 3.3. Detection of Sensitivity

The initial concentration of P.ple-sctU-O positive recombinant plasmid was established at 100 ng/μL, corresponding to a copy number of 2.62 × 10^10^ copies/reaction. It was observed that no distinct bands were visible when the template concentration was reduced to 1 × 10^−4^ ng/μL (2.62 × 10^4^ copies/reaction) following outer amplification and to 5 × 10^−8^ ng/μL (13.1 copies/reaction) after middle amplification. However, distinct bands remained visible following inner amplification, even when the template concentration was as low as 1 × 10^−9^ ng/μL (2.62 copies/reaction), indicating that the minimum detectable copy number of the constructed P.ple-sctU detection system was 2.62 copies/reaction (Figure 3).

### 3.4. Detection of Samples

All the collected large yellow croaker samples were detected by the successfully constructed nested PCR-specific primers of *P. plecoglossicida*, and the results are shown in Table 3. In the samples tested, a single positive detection was recorded for Zhoushan in 2021, with a detection rate of 7.1%. Similarly, for Zhoushan in 2022, one sample tested positive, resulting in a detection rate of 10%. In contrast, no samples from Ningde in 2022 tested positive. The positive rates of 2023 in Ningde, Ningbo, and Zhoushan were 94.4%, 84.2%, and 13%, respectively.

According to the monthly variation, positive rates in May, August, and September were observed to be less than 10%, while those in March and April approached 90%. Literature reviews and statements from local fishermen indicate that the onset months for VWND range from December to April, with March and April being periods of high incidence. The detection results of the *P. plecoglossicida* nested PCR detection method constructed in this study are consistent with the known outbreak months of VWND. As shown in Figure 4, it is evident that there is an inverse relationship between temperature and the positive rate of the samples. The observed positive rates, derived from the incidence data at various monitoring stations in Fujian Province collected by Chen from 2017 to 2018 [27], follow a similar trend but with some prolongation. This prolongation may be attributed to errors caused by the small sample sizes in August and September. Spearman correlation analysis confirmed a significant negative correlation between temperature and positive rate (r = −0.829, *p* < 0.05).

**Figure 5:**
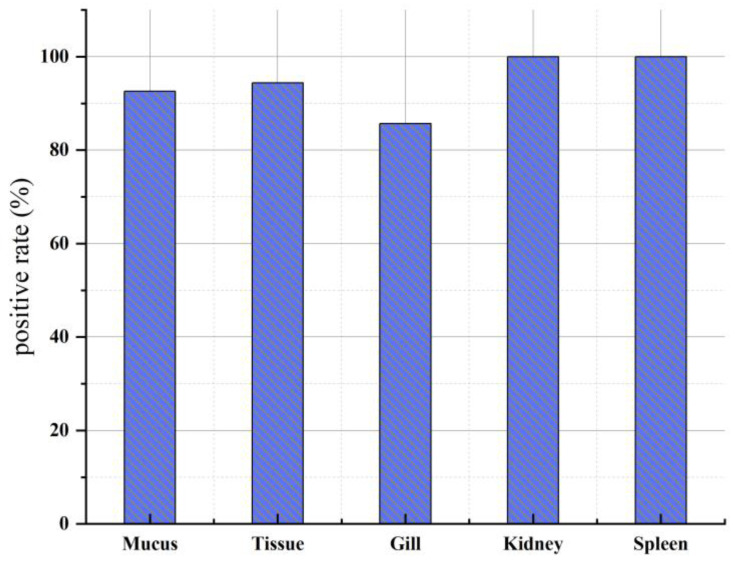
Positive rates of *P. plecoglossicida* in large yellow croaker samples from different parts.

The detection of different types of large yellow croaker samples during the high-onset period is shown in Figure 5. Among these, the spleen and kidney samples displayed the highest positive rates, reaching 100%. In comparison, the positive rate for gill samples was the lowest, at 85.7%. The muscle tissue samples and mucus samples exhibited positive rates of 94.4% and 92.6%, respectively.

## 4. Discussion

With the expansion of large yellow croaker aquaculture, various diseases have increasingly restricted the development of this industry, particularly bacterial diseases, which have incurred substantial economic losses. VWND, often undetectable in its early stages, results in high mortality rates following an outbreak. Once nodules develop in large yellow croakers, the effectiveness of drug treatments significantly diminishes [12]. Therefore, early prevention and detection have become crucial, and the importance of utilizing highly sensitive and specific pathogen detection technologies for early warning of VWND is increasingly recognized.

By optimizing the PCR reaction conditions, including the optimal annealing temperature, extension time, and the amounts of template and primer added, optimal PCR reaction conditions were established. In the specificity tests, which included nine other bacteria as the control group, it was determined that only *P. plecoglossicida* could amplify a single band with the correct length. The positive control plasmid was prepared using the amplified product of P.ple-sctU-O as the template, and a dilution gradient from 1 to 10^10^ was set for the sensitivity test. The results showed that after three rounds of nested PCR amplification, the minimum detectable copy number reached 2.62 copies/reaction, which was 10^4^ times more sensitive than a standard PCR reaction.

Zhou et al. established a *P. plecoglossicida* SYBR GreenI fluorescence quantitative PCR detection method based on the *rpoD* gene of *P. plecoglossicida* [28], achieving a sensitivity of up to 1.9 × 10^3^ copies/reaction. Based on the *gyrB* gene of *P. plecoglossicida*, Cui et al. [29] employed real-time fluorescent PCR (qRT-PCR) to detect the concentration of pathogenic bacteria in a juvenile large yellow croaker artificially infected with *P. plecoglossicida*. Li et al. [30] constructed a multiplex PCR detection method with a sensitivity of 4 ng/μL. Ding [31] designed a loop-mediated isothermal amplification technology based on the *gyrB* gene of *P. plecoglossicida* and established a real-time fluorescent LAMP method and a loop-mediated isothermal amplification combined with a lateral flow test strip for rapid detection of *P. plecoglossicida*. Mao et al. [32] reported a rapid detection of *P. putida* based on the *rpoN* gene, noting that the assay’s sensitivity of 4.8 cfu/reaction was 10 times higher than that of conventional PCR. At present, a variety of detection methods have been developed, each offering different advantages. However, due to the low content of bacteria in the early stages of the disease, it is particularly important to construct a detection method with high specificity and sensitivity. Izumi et al. [33] designed PCR primers based on the *gyrB* gene of *P. plecoglossicida*, achieving a detection limit of 0.86 CFU. This study confirmed that the nested PCR technology based on the *gyrB* sequence could be utilized for the diagnosis of BHA. Various simple detection methods have been established in the studies mentioned above, some of which allow for direct visual judgment. However, the nested PCR detection method constructed in this study offers extremely high sensitivity and provides a sufficiently sensitive method for early detection.

Meanwhile, to evaluate the practical application of the nested PCR detection system developed in this study, pathogenic samples of large yellow croaker were collected and analyzed from Zhoushan and Xiangshan in Zhejiang Province and Ningde in Fujian Province during 2021–2023. The results showed that the detection rates of the samples from Zhoushan in September 2021 and August 2022 were 7.1% and 10%, respectively. The detection rate was 0 in Ningde in July 2022, and in 2023, the detection rates were 94.4%, 84.2%, and 13% in Ningde, Ningbo, and Zhoushan, respectively. According to the results, a strong correlation was found between the detection rate of large yellow croaker samples and specific months. The detection rate of VWND in March and April was about 90%, while in other months, it was less than 10%, corroborating the results of Li et al. [5]. These results indicate that the developed detection system is not only reliable in practical applications but also easy to operate. It is anticipated that this system could be utilized for early warning and routine detection of VWND in cultured species such as large yellow croakers.

At the same time, the detection results of large yellow croaker samples from various microbial sources during the epidemic period from March to April were analyzed. It was found that gill tissue exhibited the lowest detection rate, followed by mucus and muscle tissue. Wakabayashi [34] used q-PCR technology to investigate the distribution of *P. plecoglossicida* in fish tissues. According to his study, skin and gills were identified as the initial points for bacterial invasion, with bacteria enriching in the liver, spleen, and kidney within 6 h post-infection. This observation explains why the detection rate using gill tissue is the lowest. The most plausible explanation is that most of the collected large yellow croaker samples were in the late stages of the disease, whereas the gills, serving as the primary entry points for bacterial invasion, contained higher bacterial content in the early stages, which decreased after the bacteria spread internally during the later stages. Huang et al. [35] used dual RNA-seq technology to dynamically monitor gene expression changes between bacterial pathogens and their hosts, discovering that *P. plecoglossicida* significantly accumulated in the spleen 48 h post-infection. It was found that the highest detection rates were obtained from two microbial enrichment areas, the spleen and kidney, achieving a positive rate of 100%. Among the five types of large yellow croaker samples, mucus samples are the most recommended. Firstly, mucus samples can be collected simply and with less difficulty compared to other samples. Second, these samples do not affect the normal growth of large yellow croakers, causing the least bodily harm. Finally, due to their high accuracy and sensitivity, mucus samples have proven most practical for use in field applications.

## 5. Conclusions

In this study, a highly sensitive and specific nested PCR detection system was established for detecting visceral white nodule disease in large yellow croakers caused by *P. plecoglossicida*. A triple-nested PCR system was designed based on the *sctU* gene of *P. plecoglossicida* XSDHY-P (NZ_CP031143.1) from GenBank. With the exception of *P. plecoglossicida*, no target bands could be amplified by other control bacteria, and the minimum detection limit was established at 1 × 10^−9^ ng/μL (2.62 copies/reaction). During the epidemiological survey of large yellow croakers from 2021 to 2023, it was found that the positive rate of *P. plecoglossicida* in all samples was 64.6%. The results showed that March and April were the periods of high incidence for large yellow croaker, inversely related to temperature. At the same time, the advantages and disadvantages of different samples for detection were compared, and mucus samples were considered to have the highest practical application value.

## 6. Patents

The pathogen detection method constructed in this study has applied for a Chinese invention patent (application number: 202410272186.X).

## Figures and Tables

**Table 1:** Primer sequences and product length.

Primer Name		Sequence (5′–3′)	Expected Product Length (bp)
P.ple-sctU-O	OF	CGCTACTCACTGGCTACA	795
	OR	CGACGCCTTCTTCTTCC	
P.ple-sctU-M	MF	CAGATGTTGCTGCTGTGC	326
	MR	ACCAATGCCTGCTCGTT	
P.ple-sctU-I	IF	AACGAAGGTAGCCCACA	133
	IR	GATGCCCACGGCAATAT	

O: Outer, M: Middle, I: Inner.

**Table 2:** Detailed information on sampling of *L. crocea* samples.

Sampling Date	Sampling Location	Types and Quantities of Samples		
		Surface Mucus	Tissues	Total
September 2021	Zhoushan, Zhejiang	4	10	14
July–August 2022	Ningde, Fujian	4	4	8
	Zhoushan, Zhejiang	5	5	10
March–May 2023	Ningde, Fujian	20	70	90
	Ningbo, Zhejiang	4	15	19
	Ningde, Fujian	8	15	23
Total		47	117	164

**Table 3:** Positive rates of *P. plecoglossicida* in large yellow croaker samples.

Sample Group	Surface Mucus		Tissues		Positive Rate in Total
	Detections	Positive Rate	Detections	Positive Rate	
September 2021 Zhoushan	0	0	1	10%	7.1%
July 2022 Ningde	0	0	0	0	0
August 2022 Zhoushan	0	0	1	20%	10%
March 2023 Ningde	17	85%	68	97.1%	94.4%
April 2023 Ningbo	3	75%	13	86.7%	84.2%
May 2023 Zhoushan	1	12.5%	2	13.3%	13.0%
Total	21	44.7%	85	72.6%	64.6%

## Data Availability

The original contributions presented in the study are included in the article, further inquiries can be directed to the corresponding authors.
